# Comparison of the effects of two different trigger strategies - dual (hCG + Leuprolide) *versus* hCG trigger - in antagonist non-donor IVF: a randomized controlled trial

**DOI:** 10.5935/1518-0557.20230040

**Published:** 2023

**Authors:** Neeta Singh, Aryan Kashyap, Neena Malhotra, Reeta Mahey, Richa Vatsa, Garima Patel

**Affiliations:** 1 Department of Obstetrics and Gynaecology, All India Institute of Medical Sciences, New Delhi, India

**Keywords:** dual trigger, single trigger, antagonist cycle

## Abstract

**Objective:**

Conventionally, hCG is used as a ‘faux’ LH surge to bring final oocyte maturation due to structural similarity with LH. Although GnRH agonists induce a more physiological gonadotropin surge for follicular maturation, they have been associated with luteal phase deficiency. Our aim was to assess whether adding a gonadotropin-releasing hormone agonist (GnRHa) to hCG trigger improves oocyte maturation and the number of high-grade embryos in GnRH antagonist IVF cycles.

**Methods:**

This was a single center, open-labelled, randomized controlled trial including 100 patients between 21-38 years (tubal factor, male factor, unexplained infertility, with normal ovarian reserve) undergoing IVF using the GnRH antagonist protocol. Patients were randomized to receive either the dual trigger (Leuprolide acetate 1mg + rhCG 250µg, n=50) or a single hCG trigger (rhCG 250µg, n=50). Analysis was done by ITT. Independent-t and chi-square tests were used in the comparisons of normally distributed quantitative variables and qualitative variables.

**Results:**

With similar baseline characteristics, the number of MII oocytes (7.82 *vs*. 5.92, *p*=0.003) and day-3 grade-1 embryos (4.24 *vs*. 1.8, *p*<0.001) and consequently, number of embryos cryopreserved (2.68 *vs*. 0.94, *p*<0.001) were significantly higher in the dual trigger group. However, the fertilization (91.82% *vs*. 88.51%, *p*=0.184) and clinical pregnancy rates between the two groups (21% *vs*. 19.6%, *p*=0.770) were comparable. Serum LH levels 12 hours post trigger were high in the dual trigger group (46.23mIU/ml vs. 0.93mIU/ml, *p*<0.0001).

**Conclusions:**

This study found that the addition of GnRHa to hCG trigger leads to improved embryological outcomes and the possibility of cryopreserving surplus embryos, thereby increasing cumulative live births.

## INTRODUCTION

Follicle development and dominant follicle selection are tightly regulated by pituitary gonadotropins and intraovarian regulators (Orisaka *et al*., 2021). Finally, ovulation is preceded by a surge in gonadotropins (both LH and FSH), which leads to final oocyte maturation, resumption of meiosis and luteinization of the granulosa cells (Haas *et al*., 2020). In ovarian stimulation protocols in IVF, final oocyte maturation and resumption of meiosis is brought about by a “trigger”, that is given 35-37 hours before oocyte retrieval.

Traditionally, human chorionic gonadotropin (hCG) has been used as “faux” LH surrogate to induce final oocyte maturation and meiosis. However, it is now well understood that hCG and LH differ in the mechanism of inducing final oocyte maturation at a molecular level (Riccetti *et al*., 2017; Casarini *et al*., 2012). Even though hCG binds to the same receptor as LH to produce a response that mimics the mid-cycle LH surge, there is a major structural difference between the two hormones that lends critical difference to their pharmacokinetics and clearance.

Gonen *et al.* (1990) showed that GnRH agonists could also be used to trigger final oocyte maturation in GnRH antagonist cycles, by stimulating an LH and FSH surge, which was more physiological as compared to hCG trigger. FSH surge is now known to be of importance in the expression of an adequate complement of LH receptors on the granulosa layer (Orvieto, 2015). Studies from various authors have shown that the number of oocytes retrieved, the number of MII oocytes and the number of top-quality embryos were either comparable or favored the GnRH agonist trigger (Fauser *et al*., 2002; Humaidan *et al*., 2005; Kolibianakis *et al*., 2005; Erb *et al*., 2010). However, there is a significant quantitative reduction in gonadotropins released from the pituitary after a GnRH agonist trigger, leading to corpus luteum deficiency, luteolysis and luteal phase deficiency, culminating in lower pregnancy rates and higher rates of early pregnancy losses (Humaidan *et al*., 2005).

This led to the development of the concept of a ‘dual trigger’, where a GnRH agonist is used together with hCG as a trigger to achieve final oocyte maturation. Studies conducted by different authors in patients with diminished ovarian reserve (DOR) (Chern *et al*., 2020; Lin *et al.*, 2019; Maged *et al*., 2021) as well as studies in normal responders (Haas *et al*., 2020; Alleyassin *et al*., 2018; Ali *et al*., 2020) comparing patients receiving dual trigger versus hCG trigger have found better results in terms of clinical pregnancy, live birth and fertilization rates in the dual trigger group. By adding a GnRH agonist to hCG, the ‘dual trigger’ leads to better oocyte yield, a greater number of MII oocytes retrieved, and a greater number of good quality embryos. GnRH agonists cause a surge in both FSH and LH that closely mimics the natural mid-cycle gonadotropin surge, whereas hCG also provides an added benefit by providing luteal phase support, thereby increasing clinical pregnancy and live birth rates in patients undergoing IVF. Incited by the aforementioned observations, we performed a prospective, randomized controlled trial in normal responders, comparing GnRH antagonist down-regulated cycles triggered with hCG versus dual trigger (GnRH agonist + hCG) 35-37 hours before oocyte retrieval.

## MATERIALS AND METHODS

This prospective, randomized non-blinded controlled trial was carried out at the ART Center, Department of Obstetrics and Gynecology, of a tertiary care center from January 2020 to August 2021. After obtaining ethical clearance, a total of 100 women were enrolled in the study. With informed consent, women between the ages of 21 and 38 years with either tubal factor, male factor or unexplained infertility, with a normal ovarian reserve, were included in the study; women with a thin endometrium, prior history of uterine anomaly/surgery, PCOS, poor ovarian reserve [antral follicle count (AFC)<5 and/or serum anti-Müllerian hormone (AMH) levels <1.2 ng/ml] and known medical comorbidities such as diabetes/hypertension were excluded from the study. Prior to recruitment into an IVF cycle, baseline hormone profile [day-2/3 luteinizing hormone (LH), follicle stimulating hormone (FSH), prolactin (Prl), thyroid stimulating hormone (TSH) and AMH], 4-D ultrasound for uterine cavity assessment, day-2-5 AFC and day-16 endometrial thickness were assessed. Randomization was performed using a computer-generated random number table in a 1:1 ratio at the time of administration of trigger to either receive the hCG trigger or the dual (hCG + Leuprolide) trigger. Neither the patients nor the investigators of the study were blinded.

Sample size was calculated based on a previous study by Mahajan *et al*. (2016) and presuming similar outcomes for the number of good quality day-3 embryos for 80 percent power at a 5 percent level of significance, yielding a sample size of 180 patients. However, due to the ongoing COVID-19 pandemic, we decided to conduct an interim analysis once a total of 100 patients were recruited.

Controlled ovarian stimulation with flexible antagonist protocol was started for patients meeting the inclusion criteria. Baseline USG was performed on day 2 of the cycle. Recombinant FSH (Gonal F, Merck Serono, Mumbai, India) was started from day 2 or day 3 of the menstrual cycle. Gonadotropin dose was decided based on patient age, BMI, and ovarian reserve parameters. Ultrasonographic assessment by transvaginal sonography was started from day 5 of stimulation. GnRH antagonist cetrorelix acetate (Injection Cetrotide, Merck Serono Specialties Pvt. Ltd., India) 250ug was added on the 6^th^ day of the menstrual cycle (fixed-dose regimen) or when the follicular size was 14mm (flexible protocol), and was continued until the day of the trigger. Oocyte maturation was achieved with a trigger when at least three leading follicles of size ≥ 18 mm were seen on TVS.

On the day of trigger administration, the patients were randomized to either of the two groups of the study. Group A (n=50) received the single trigger with a subcutaneous injection of r-hCG (Inj Ovitrelle, Merck Serono, Mumbai, India) 250 µg. Group B (n=50) received the dual trigger with an injection of Leuprolide (Inj. Lupride, Sun Pharmaceuticals Industries Ltd.) 1 mg subcutaneously simultaneously with an injection of r-hCG (Inj. Ovitrelle, Merck Serono, Mumbai, India) 250 µg subcutaneously. Blood samples were drawn 12 hours later to evaluate LH levels.

Oocyte retrieval will be performed 36-37 h post trigger, followed by in-vitro sperm insemination. Post fertilization, 1 or 2 embryos were transferred between days 3 and 5, depending on patient response and embryo quality. All embryo transfers were performed by a single fellowship-trained senior faculty under abdominal ultrasonography guidance using an embryo catheter. Both groups received luteal phase support with intramuscular injections of progesterone 100 mg once daily or micronized progesterone vaginal pessary 600mg daily in two divided doses. Pregnancy tests were considered positive when positive serum hCG levels (> 30 IU/ml) were detected 14 days after embryo transfer. Clinical pregnancy was defined by a viable intrauterine gestation in the 6-week scan. Multiple gestational sacs were counted as one clinical pregnancy. The clinical pregnancy rate was calculated as the number of clinical pregnancies per embryo (Zegers-Hochschild *et al*., 2017).

### Statistical analysis

Data analysis was carried out using statistical software STATA version 20.0. Continuous variables were tested for normality using the appropriate statistical tests. Comparison of mean values was performed via Student’s t-test. Categorical variables were presented as frequencies and percent values. Frequency data for two categorical variables were compared using Chi-square/Fisher’s exact test. For all statistical tests, a two-sided probability of *p*<0.05 was considered for statistical significance.

## RESULTS

**Figure 1:**
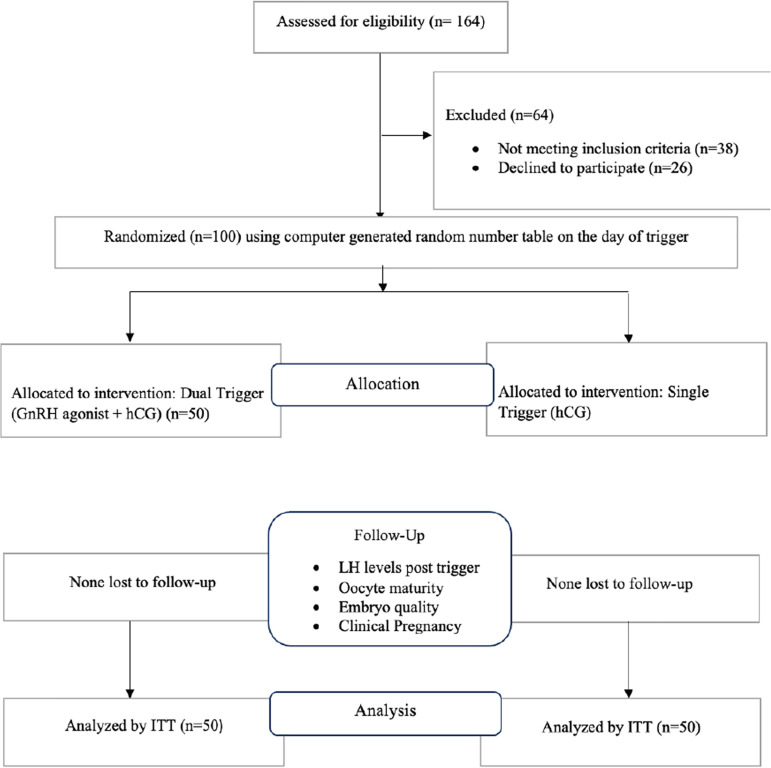
CONSORT FLOW of participants.

A total of 100 patients were enrolled and randomized to receive either the dual (n=50) or the hCG trigger (n=50) (Fig. 1). Baseline characteristics such as age, BMI, baseline hormone profile and ovarian reserve parameters were similar between the two groups (*p>*0.05) (Table 1). Similarly, the ovarian stimulation characteristics were similar between the two groups (Table 2).

**Table 1:** Baseline characteristics between the two groups.

Baseline characteristics	Dual trigger group (n=50)	Single trigger group (n=50)	*p*-value
Age (years)	30.98±4.340	30.88±3.696	0.902
BMI (kg/m^2^)	24.37±3.59	24.60±2.64	0.702
LH	4.38±1.89	4.17±1.93	0.590
FSH	5.82±1.7	5.54±1.77	0.419
AMH	2.75±0.87	2.79±0.84	0.796
Total AFC	12.2±2.02	12.04±2.36	0.717

**Table 2:** Ovarian stimulation characteristics between the two groups.

Ovarian stimulation characteristics	Dual trigger group (n=50)	Single trigger group (n=50)	*p*-value
Starting dose of FSH (IU)	241.5±64.8	258.18±59.39	0.183
Starting dose of HMG (IU)	123±65.63	121.5±64.09	0.908
Total dose of FSH	2371.3±596.17	2351.4±585.06	0.867
Total dose of HMG	758.98±684.66	943.5±742.7	0.908
Days of stimulation	9.58±1.2	9.74±1.29	0.522
E2 levels on day of trigger	2,539.86±252.80	2,765.86±271.29	0.238
P4 levels on day of trigger	1.21±0.54	1.17±0.47	0.740

**Figure 2:**
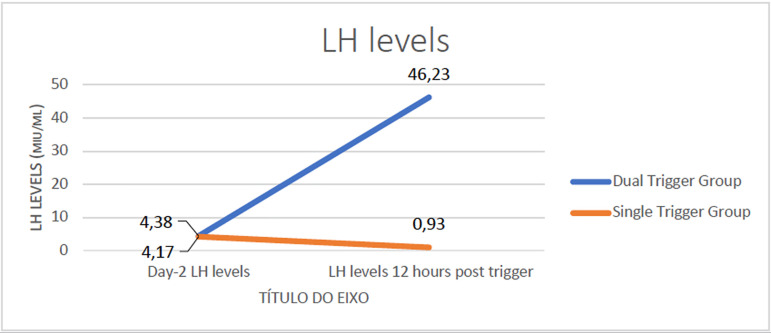
LH levels 12 hours post trigger.

Serum LH levels measured 12 hours post trigger were higher in the dual trigger group as compared to the hCG group (46.23 *vs*. 0.93 mIU/ml, *p*<0.0001, Fig. 2). This is an expected outcome, since it is known that the GnRH agonist in the dual trigger combination brings about a surge of LH, which mimics the mid-cycle gonadotropin surge and is, hence, a more physiological trigger.

Even though the total number of oocytes retrieved were similar the two groups (9.48±3.96 *vs*. 8.2±3.19, *p*=0.078), the number of MII oocytes were significantly higher in the dual trigger group than the hCG trigger group (7.82±3.24 *vs*. 5.92±2.93, *p*=0.003). Subsequently, the number of grade-1 embryos (4.24±2.24 *vs*. 1.8±1.78, *p<*0.001) and the number of embryos cryopreserved (2.68±2.22 *vs*. 0.94±1.38, *p<*0.001) were also significantly higher in the dual trigger group than in the hCG trigger group. Though the CPR was higher in the dual trigger group, the difference relative to the hCG trigger group was not statistically significant (21% *vs*. 19.6%, *p*=0.770) (Table 3). None of the participants in either of the groups developed OHSS.

**Table 3:** IVF outcomes.

	Dual trigger group (n=50)	Single trigger group (n=50)	*p*-value
Oocytes retrieved (n)	9.48±3.96	8.2±3.19	0.078
OCC grade 1 (n)	4.5±2.21	3.66±2.12	0.055
OCC grade 2 (n)	4.3±1.81	4.02±1.65	0.420
MII oocytes (n)	7.82±3.24	5.92±2.93	0.003
2PN (n)	7.18±2.99	5.24±2.74	0.001
Fertilization rates	91.82%	88.51%	0.184
Embryos (n)	7.04±2.99	4.96±2.81	0.001
Grade-I embryos	4.24±2.24	1.8±1.78	<0.001
Embryos frozen(n)	2.68±2.22	0.94±1.38	<0.001
Clinical Pregnancy Rate	21%	19.6%	0.770

## DISCUSSION

The results of our study validate the hypothesis that by adding a GnRH agonist to the hCG trigger, the dual trigger does help achieve better oocyte maturation, as evidenced by a greater number of MII oocytes retrieved in the dual trigger group. We observed a statistically significant increase in the number of mature MII oocytes, day-1 2PN, total number of embryos, and number of grade-1 embryos in the dual trigger group. Consequently, the number of embryos cryopreserved was also significantly greater in the dual trigger group.

Our study included women with a normal ovarian reserve (AMH >1.2 ng/ml and AFC between 5-25), with no history of uterine surgery. Women with diminished ovarian reserve or at a high risk of OHSS were excluded, since these were variables associated with adverse IVF outcomes. This was in concurrence with previous studies by Lin *et al*. (2013) and Seval *et al*. (2016), which also excluded women with occult ovarian failure (day-3 FSH ≥ 10 IU/L or AMH levels ≤1 ng/ml). Similarly, in a trial by Haas *et al*. (2020), only women with AMH >1 ng/ml and AFC of 6-20 were included, thus excluding high responders and DOR patients.

The current study is one of the few to study LH levels 12 hours after the administration of the trigger. The prospective clinical trial by Decleer *et al*. (2014) made an attempt to study the evolution of LH and FSH following oocyte maturation. Significantly higher FSH and LH levels were reported in the dual trigger group on the day prior to oocyte retrieval, with FSH levels remaining higher for a longer time. Both LH and FSH play an important role in oocyte maturation, and the additional FSH surge is thought to promote oocyte meiosis, cumulus expansion and the release of various proteolytic enzymes that play a central role in ovulation. No LH surge was observed in the hCG group, whereas an evident LH peak (up to 60IU/L) was observed in the combined trigger (Decleer *et al*., 2014). This is an expected finding that supports the hypothesis that the more physiological gonadotropin surge associated with the dual trigger might be the reason for greater MII oocyte yield and better embryo quality in the dual trigger group.

Though the clinical trial by Alleyassin *et al*. (2018) found no significant increase in the number of MII oocytes (9.52±6.07 *vs*. 8.71±5.11, *p*=0.42), more compelling evidence was produced from recent randomized controlled trials by Ali *et al*. (2020) and Haas *et al*. (2020), where the authors found significantly higher number of MII oocytes retrieved from the dual trigger group (10.78±6.758 vs. 8.48±4,0 *p*=0.01 and 10.3 *vs*. 8.6, *p*=0.009; respectively) as compared to the standard trigger (hCG) alone. We also observed similar improvements in the number of MII oocytes in the dual trigger group, which directly translates into greater number of good quality embryos. The clinical trial by Decleer *et al*. (2014) found that the proportion of patients with at least one top quality embryo was higher in the dual trigger group when compared with the single trigger group (73.8% *vs*. 47.5%, *p*=0.001). Further, Alleyassin *et al.* (2018) also demonstrated that, despite having comparable number of MII oocytes, the dual trigger group had a significantly greater number of high quality embryos than the hCG-triggered group (6.4±3.8 *vs*. 5.03±3.4, *p*=0.04). More recently, the clinical trial by Haas *et al*. (2020) also showed a significant increase in the number of blastocysts (3.9 *vs*. 2.9, *p*=0.01) and number of top quality blastocysts (2.2 *vs*. 1.4, *p*=0.001) in the dual trigger group. These findings corroborate with those of the current study, where we found a significant increase in the number of grade-1 day-3 embryos (4.24±2.24 *vs*. 1.8±1.78, *p*<0.001) in the dual trigger group.

In the current study, a significantly higher numbers of embryos were cryopreserved in the dual trigger group than in the single trigger group (2.68±2.22 *vs*. 0.94±1.38, *p*<0.001). The importance of this outcome lies in the fact that greater number of embryos cryopreserved are likely to improve the cumulative live birth rate. Patients with greater number of cryopreserved embryos who do not conceive in a fresh cycle, can be planned for a frozen embryo transfer in the next cycle, which markedly reduces the costs incurred by the patient. Decleer *et al*. (2014) found that even though the number of embryos cryopreserved were not statistically different, the number of patients with embryos left for cryopreservation were significantly higher in the dual trigger group (54.1% *vs*. 33.6%, *p*=0.04).

There was no significant difference in clinical pregnancy rates between the dual and single trigger groups (21% *vs*. 19.6%, *p*=0.7). Despite the higher number of MII oocytes and grade-1 embryos in the dual trigger group, the reproductive outcome in terms of clinical pregnancy rate was similar between the two groups, as high LH levels has been shown to decrease endometrial receptivity (Ali *et al*., 2020). Whether this outcome is a result of high LH levels negatively impacting endometrial receptivity or a smaller sample size, is a matter that needs further introspection and evidence from well-designed, multicenter clinical trials that follow the outcomes to verify the effects on cumulative live birth rates. However, in a recent randomized controlled trial by Haas *et al*. (2020), the authors found pregnancy outcomes in terms of total implantation rate (43.2% *vs*. 22.8%, *p*=0.03), live birth rate per transfer (36.2% *vs*. 22%, *p*=0.02) and clinical pregnancy rate per patient (56.8% *vs*. 37.3%, *p*=0.02) to be significantly higher in the dual trigger group.

It is of due importance to note that the dose of GnRH agonist and hCG in the dual trigger has not been standardized and no fixed dosage of the same exists for dual trigger (Table 4). In tandem with our study, Ali *et al*. (2020) triggered final oocyte maturation with 250 µg of recombinant hCG along with 1mg leuprolide acetate in the dual trigger group, whereas the hCG trigger group received 250 µg of recombinant hCG. Thus, it is evident that the dose of GnRH agonist to be used in dual trigger has not been standardized to date. Further research is required in this aspect of dual trigger, so that a reference standard dose of GnRH agonist along with hCG can be used to attain maximum benefits in terms of both embryological and reproductive outcomes.

**Table 4:** Comparison of study design, sample size, triggers and ART procedure in different studies.

Author	Study Design	Participants			Trigger		ART
		Total	Group		Group		
			Intervention	Reference	Intervention	Reference	
Schachter *et al*., 2008	Prospective RCT	221	105	106	Triptorelin 0.2mg + hCG 5,000IU	hCG 5,000IU	IVF and IVF-ICSI
Lin *et al*., 2013	Retrospective Cohort Study	376 (378 cycles)	191	187	Triptorelin 0.2mg + rhCG 250µg	rhCG 250µg	IVF and IVF-ICSI
Decleer *et al*., 2014	Prospective RCT	120	61	59	Triptorelin 0.2mg + hCG 5,000IU	hCG 5,000IU	IVF-ICSI
Mahajan *et al*., 2016	Prospective RCT	76	38	38	Leuprolide 1mg + hCG 5,000IU	hCG 10,000IU	IVF-ICSI
Seval *et al*., 2016	Retrospective Case Control	156	84	72	Leuprolide 0.5mg + rhCG 250µg	rhCG 250µg	IVF and IVF-ICSI
Alleyassin *et al*., 2018	Prospective RCT	126	63	63	Triptorelin 0.2mg + hCG 5,000IU	hCG 10,000IU	ICSI
Ali *et al*., 2020	Prospective RCT	160	80	80	Leuprolide 1mg + rhCG 250µg	rhCG 250µg	ICSI
Haas *et al.*, 2020	Prospective RCT	155	77	78	Buserelin 0.5mg + 10,000IU hCG	hCG 10,000IU + Placebo (normal saline)	IVF and IVF-ICSI
Current study	Prospective RCT	100	50	50	Leuprolide 1mg + rhCG 250µg	rhCG 250µg	IVF and IVF-ICSI

In conclusion, the current study highlights the favorable outcomes associated with triggering final oocyte maturation with the dual trigger. With the only exception of high responders, the dual trigger is an effective strategy to improve the outcomes of ART in women undergoing antagonist downregulated cycles. Even though our study showed comparable clinical pregnancy rates between the two groups, the better embryo quality and the greater number of cryopreserved embryos associated with the dual trigger may dramatically improve cumulative live births. The strength of the study lies in the fact that it was a randomized trial and in its strict inclusion and exclusion criteria, which made it possible to remove all confounding variables of adverse IVF outcomes. But due to paucity of time, and the major impact of COVID-19 on ART services, outcomes were followed to clinical pregnancy rates only. Nevertheless, we recommend that large, robust randomized clinical trials comparing dual trigger with standard hCG trigger be organized to follow the reproductive outcomes of women undergoing both fresh and frozen embryo transfers in subsequent cycles, thus comparing outcomes in terms of cumulative pregnancy rate.
